# 

**Published:** 1946

**Authors:** 


### CONTENTS.

| | Page |
|---|---|
| The Thirty-third Long Fox Memorial Lecture: English Doctors from Smollett to Trollope. By E. WATSON-WILLIAMS, CH.M. .. | 89 |
| Symptomless Hyperpiesia in Dockyard Workers. By J. M. NAISH, M.A., M.B., B.Ch., M.R.C.P. .. .. .. .. | 106 |
| Pituitary Cachexia Treated with Corticotrophic Hormone. By R. E. HEMPHILL, M.A., M.D., D.P.M. .. .. .. .. | 116 |
| Reviews of Books .. .. .. .. .. .. | 119 |
| Meetings of Societies .. .. .. .. .. | 121 |
| Local Medical Notes .. .. .. .. .. | 121 |

